# Supplementary material for: Alternative isoforms of KDM2A and KDM2B lysine demethylases negatively regulate canonical Wnt signaling
Source: PLoS One. 2020 Oct 26;15(10):e0236612. doi: 10.1371/journal.pone.0236612 (PMC7588095; doi:10.1371/journal.pone.0236612)
Supplement: S1 Table — (DOCX) [file pone.0236612.s001.docx]

| **name** | **assay** | **target** | **sequence (5´to 3´)** |
| --- | --- | --- | --- |
| **Q-PCR primers** | | | |
| F1 | Q-RT-PCR | human *GAPDH* exon 7 | TGCACCACCAACTGCTTAGC |
| R1 | Q-RT-PCR | human *GAPDH* exon 8 | GCCATCCACAGTCTTCTGGG |
| F2 | Q-RT-PCR | human *HPRT1* exon 3 | TCTTTGCTGACCTGCTGGATTAC |
| R2 | Q-RT-PCR | human *HPRT1* exon 6 | GTCTGCATTGTTTTGCCAGTGTC |
| F3 | Q-RT-PCR | human *RPL32* exon 1 | CTCAGACCCCTTGTGAAGCC |
| R3 | Q-RT-PCR | human *RPL32* exon 2 | TTGCTTCCATAACCAATGTTGG |
| F4 | Q-RT-PCR | human *AXIN2* exon 3 | CTGACGGATGATTCCATGTCC |
| R4 | Q-RT-PCR | human *AXIN2* exon 4 | GGGAAATGAGGTAGAGACACTTGG |
| F5 | Q-RT-PCR | human *CYCLIN D1* exon 3 | AACAGATCATCCGCAAACACG |
| R5 | Q-RT-PCR | human *CYCLIN D1* exon 4 | GTTGTTGGGGCTCCTCAGG |
| F6 | ChIP-Q-PCR | human *AXIN2* promoter | GGATCAATGGTGAGTGCCG |
| R6 | ChIP-Q-PCR | human *AXIN2* promoter | AAATAGCCGGCCTGCCA |
| F7 | ChIP-Q-PCR | human *CYCLIN D1* promoter | CCGGGCTTTGATCTTTGCT |
| R7 | ChIP-Q-PCR | human *CYCLIN D1* promoter | AGGCTCCAGGACTTTGCAACT |
| F8 | ChIP-Q-PCR | human negative control region | AACCTGTTAATGGGCGCGTA |
| R8 | ChIP-Q-PCR | human negative control region | GCGGGAGTTTAGAGCCAGAGA |
| **cloning primers** | | | |
| F9 | cloning | human *KDM2A* exon 1 | GGAATTCCATGGAACCCGAAGAAGAAAGGATTC |
| R9 | cloning | human *KDM2A* exon 21 | GCTCTAGAGCTTAGCTGATCTTCTGTATCAGCTTC |
| F10 | cloning | human *KDM2A* exon 14 | GGAATTCCATGAAACCAGCTCCACGGTTAACA |
| R10 | cloning | human *KDM2A* exon 21 | GCTCTAGAGCTTAGCTGATCTTCTGTATCAGCTTC |
| F11 | cloning | mouse *Kdm2b* exon 1 | GGAATTCCATGGAGGCAGAGAAAGACTCTGG |
| R11 | cloning | mouse *Kdm2b* exon 21 | GCTCTAGACATACTTGTCCTGGAACTAACTTAG |
| F12 | cloning | mouse *Kdm2b* exon 1b | GGAATTCCATGGCCATGTCCGTGAGCGCC |
| R12 | cloning | mouse *Kdm2b* exon 21 | GCTCTAGACATACTTGTCCTGGAACTAACTTAG |
| F13 | cloning | mouse *Tcf7l1* exon 1 | GGAATTCCATGCCCCAGCTCGGTGGTGGC |
| R13 | cloning | mouse *Tcf7l1* exon 13 | GCTCTAGAGCTTAGTGGGCAGACTTGGTGACCAA |
| F14 | cloning | human *AXIN2* promoter region 1 | CGCGTACCTCCCTTTCAGGAC |
| R14 | cloning | human *AXIN2* promoter region 1 | GCTCATCTGAACCTCCTCTCTG |
| F15 | cloning | human *AXIN2* promoter region 2 | CCGTGCTAGGGAAAGCAGCTTG |
| R15 | cloning | human *AXIN2* promoter region 2 | GCTCATCTGAACCTCCTCTCTG |
| **mutagenesis primers** | | | |
| F16 | mutagenesis | human KDM2A mutEcoRI | ATTTTCAAGAACTCTGATGGACTCGG |
| R16 | mutagenesis | human KDM2A mutEcoRI | CAGAGGATCTCTCAAGCCACCCC |
| F17 | mutagenesis | human KDM2A mutJmjC | GACTTCGCTGTGGCCTTTGGTGG |
| R17 | mutagenesis | human KDM2A mutJmjC | AGTATAGCAGCCTCGAACACTCATT |
| F18 | mutagenesis | human KDM2A mutCXXC | TGTCCTCCGACAGTGCTTGGCAC |
| R18 | mutagenesis | human KDM2A mutCXXC | CAGGACTGCGCCATGCGTCCAG |
| F19 | mutagenesis | human KDM2A mutPHD | CTCGCTGGAGAGGTGGATCAGAAT |
| R19 | mutagenesis | human KDM2A mutPHD | GGAAGCTGTGACTGAGTGAGGCA |
| F20 | mutagenesis | human KDM2A mutHP1motif | CTCACTGCCACGGCACAGAGGC |
| R20 | mutagenesis | human KDM2A mutHP1motif | GTACGATCCCCGGATCTTGGCTT |
| F21 | mutagenesis | mouse KDM2B mutJmjC | GACTTCGCCATTGCCTTTGGAGG |
| R21 | mutagenesis | mouse KDM2B mutJmjC | AGTGAAACAGCCCTTCACGCTCAT |
| F22 | mutagenesis | mouse KDM2B mutCXXC | CCGAGGCCGCCCTGCGGACG |
| R22 | mutagenesis | mouse KDM2B mutCXXC | CCTTGCGGGCTCGCGTCCGG |
| F23 | mutagenesis | mouse KDM2B mutPHD | GAAGCCTCCATCGCCAACGAGAT |
| R23 | mutagenesis | mouse KDM2B mutPHD | CATGAGCATGAGGTTAAACTTGCCT |
