# Supplementary figures and images for: Alternative isoforms of KDM2A and KDM2B lysine demethylases negatively regulate canonical Wnt signaling
Source: PLoS One. 2020 Oct 26;15(10):e0236612. doi: 10.1371/journal.pone.0236612 (PMC7588095; doi:10.1371/journal.pone.0236612)

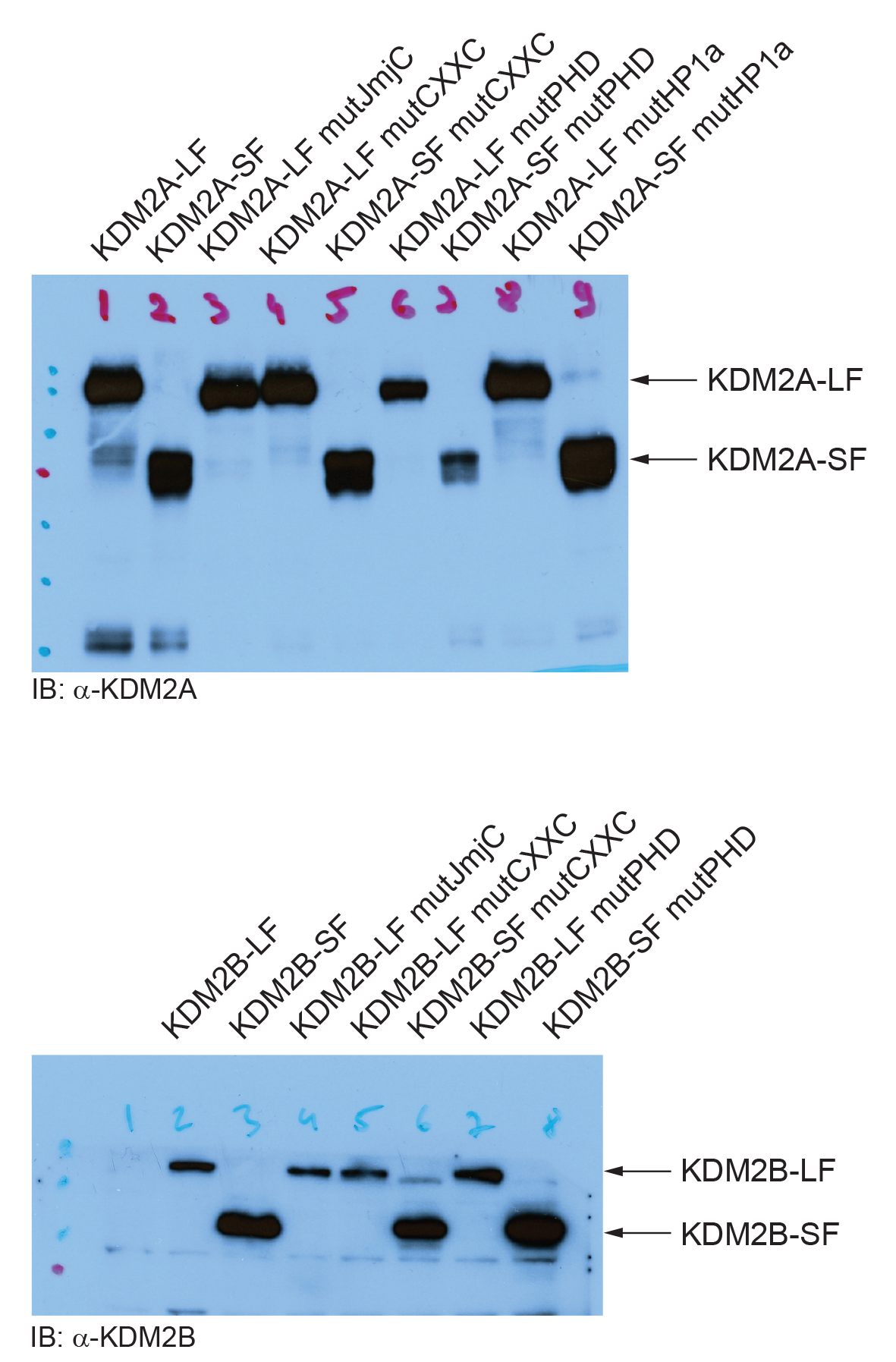

Supplement: S1 Fig — The nuclear extracts from the HEK293T cells transfected with the wild type and mutant pCS2-KDM2A/B constructs were analyzed by western blot using the KDM2A or KDM2B antibodies. (TIF) [file pone.0236612.s002.tif]

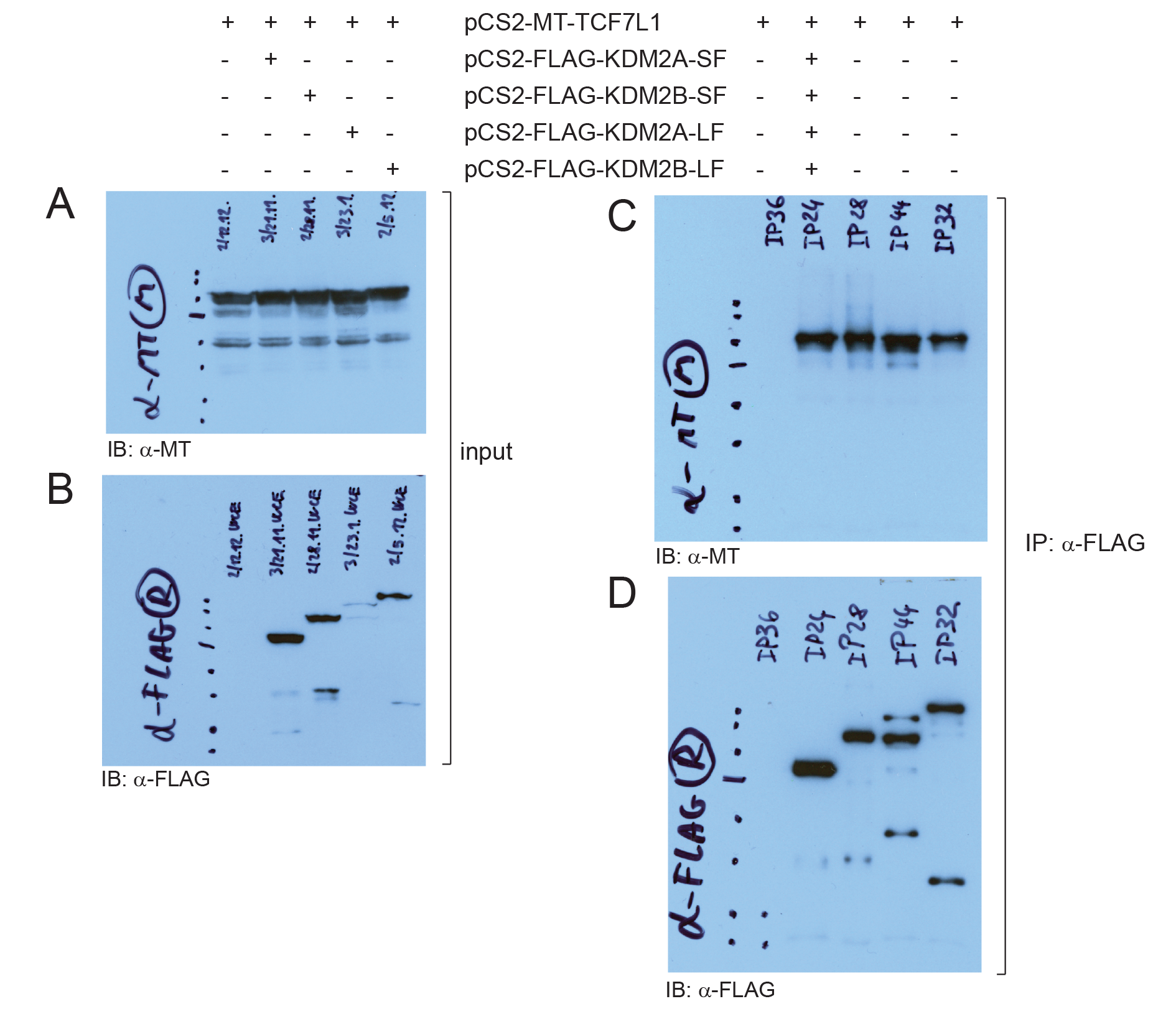

Supplement: S2 Fig — A. The inputs (50 μg) of the whole cell extracts analyzed by western blot using the anti-myc tag antibody to show the expression of the myc-tagged TCF7L1 protein in the transfected HEK293T cells. B. The inputs (50 μg) of the whole cell extracts analyzed with the anti-FLAG antibody to show the expression of the FLAG-tagged KDM2A/B proteins. C. One third of the immunoprecipitate analyzed with the anti-myc antibody to show co-immunoprecipitation of the myc-tagged TCF7L1 protein with the KDM2A/B proteins. D. One third of the immunoprecipitate analyzed with the anti-FLAG antibody to show the immunoprecipitated KDM2A/B proteins. (TIF) [file pone.0236612.s003.tif]
